# Dalcetrapib and anacetrapib increase apolipoprotein E-containing HDL in rabbits and humans

**DOI:** 10.1016/j.jlr.2022.100316

**Published:** 2022-11-19

**Authors:** Mathieu R. Brodeur, David Rhainds, Daniel Charpentier, Marie Boulé, Téodora Mihalache-Avram, Mélanie Mecteau, Geneviève Brand, Valérie Pedneault-Gagnon, Annik Fortier, Eric J. Niesor, Eric Rhéaume, Cyrille Maugeais, Jean-Claude Tardif

**Affiliations:** 1 Montreal Heart Institute, Montreal, Quebec, Canada; 2 Montreal Health Innovations Coordinating Center, Montreal, Quebec, Canada; 3 F. Hoffmann-La Roche Ltd., Basel, Switzerland; 4 Faculty of Medicine, Université de Montréal, Montreal, Quebec, Canada

**Keywords:** CETP, cholesteryl ester transfer protein inhibitors, apolipoprotein composition, low-density lipoprotein receptor, atorvastatin, dal-PLAQUE-2 study, large HDL particles, reverse cholesterol transport, hepatic cells, lipoprotein metabolism, 2D-NDGGE, 2D-nondenaturing gradient gel electrophoresis, ADCY9 gene, adenylate cyclase type 9 gene, apo A-I, apolipoprotein A-I, apo E, apolipoprotein E, CAD, coronary artery disease, CE, cholesteryl ester, CETP, cholesteryl ester transfer protein, CETPi, CETP inhibitor, FPLC, fast protein liquid chromatography, LDLr, LDL receptor, LPS, lipopolysaccharide, RCT, reverse cholesterol transport

## Abstract

The large HDL particles generated by administration of cholesteryl ester transfer protein inhibitors (CETPi) remain poorly characterized, despite their potential importance in the routing of cholesterol to the liver for excretion, which is the last step of the reverse cholesterol transport. Thus, the effects of the CETPi dalcetrapib and anacetrapib on HDL particle composition were studied in rabbits and humans. The association of rabbit HDL to the LDL receptor (LDLr) in vitro was also evaluated. New Zealand White rabbits receiving atorvastatin were treated with dalcetrapib or anacetrapib. A subset of patients from the dal-PLAQUE-2 study treated with dalcetrapib or placebo were also studied. In rabbits, dalcetrapib and anacetrapib increased HDL-C by more than 58% (*P* < 0.01) and in turn raised large apo E-containing HDL by 66% (*P* < 0.001) and 59% (*P* < 0.01), respectively. Additionally, HDL from CETPi-treated rabbits competed with human LDL for binding to the LDLr on HepG2 cells more than control HDL (*P* < 0.01). In humans, dalcetrapib increased concentrations of large HDL particles (+69%, *P* < 0.001) and apo B-depleted plasma apo E (+24%, *P* < 0.001), leading to the formation of apo E-containing HDL (+47%, *P* < 0.001) devoid of apo A-I. Overall, in rabbits and humans, CETPi increased large apo E-containing HDL particle concentration, which can interact with hepatic LDLr. The catabolism of these particles may depend on an adequate level of LDLr to contribute to reverse cholesterol transport.

The atheroprotective function of HDL resides in multiple properties. One of them consists in the removal of excess cholesterol present in peripheral tissues and its transport to the liver for biliary excretion (1). This process requires a range of HDL subclasses involved at each step of this process called reverse cholesterol transport (RCT). Thus, drugs targeting proteins implicated in HDL remodeling have been developed and tested in clinical studies.

Cholesteryl ester transfer protein (CETP) is a plasma glycoprotein with a molecular mass of 74 kDa, which is primarily secreted by the liver (2). CETP activity is involved in the modulation of HDL-C level by mediating the transfer of cholesteryl esters (CEs) from HDL to apo B-containing lipoproteins (2, 3). Accordingly, numerous studies have demonstrated that CETP inhibition increases HDL-C and generates larger spherical HDL particles (4, 5, 6, 7, 8). Although apolipoprotein A-I (apo A-I) remains the principal apolipoprotein of mature HDL, it was shown that larger HDL particles also contain apolipoprotein E (apo E) (9). It was demonstrated, in animals (6) and humans (10), that HDL modified by the CETP inhibitor (CETPi) dalcetrapib are enriched in apo E. However, the precise apolipoprotein composition and function of these particles remain unclear and require further characterization. Moreover, it was also reported that anacetrapib, another CETPi, increases plasma apo E concentration, but this higher level was not ascribed to a specific lipoprotein class (11).

Apo E is a 34 kDa glycoprotein produced and secreted by a wide range of tissues (12), but the plasma apo E predominantly originates from the liver (13). Circulating apo E plays a pivotal role in lipoprotein metabolism since it was found associated with several classes of lipoproteins, including apo B-containing chylomicrons, VLDL, their remnants (intermediate density lipoproteins), and large HDL (9). Apo E is a bona fide ligand of LDL receptor (LDLr) family members and is consequently involved in the hepatic and extrahepatic uptake of lipoproteins (14, 15). Accordingly, it was demonstrated that the LDLr mediates the cellular uptake of apo E-enriched HDL (16, 17). Thus, an increase of these particles induced by CETP inhibition may contribute to RCT by generating a particle able to deliver cholesterol coming from the peripheral tissues to the liver via the LDLr. Interestingly, it was demonstrated that administration of statins, which increase LDLr, reduce the plasma concentration of apo E (18, 19, 20). Thus, simultaneous administration with statins could influence apo E-enriched HDL formation and modulate the effects of CETPi.

Following initial disappointing clinical results with CETPi (21, 22), it was recently demonstrated that anacetrapib reduced cardiovascular events by 9% in the REVEAL trial (23). Moreover, given that the effect of dalcetrapib on cardiovascular outcomes appears to be influenced by genotypes at the SNP rs1967309 in the *ADCY9* (adenylate cyclase type 9) gene (24, 25), a pharmacogenetics-guided phase 3 trial was initiated with this agent (dal-GenE, NCT02525939) (24, 25, 26). Although the trial was negatively impacted by the COVID-19 pandemic and did not meet the primary endpoint, the results validate the interaction between dalcetrapib treatment and AA genotype at variant rs1967309 in the *ADCY9* gene (27). This strengthens the importance of further evaluating the impact of dalcetrapib and anacetrapib on lipoprotein composition and function. Thus, the present study was designed to *1*) determine the effects of dalcetrapib and anacetrapib on the composition and function of large HDL particles in vivo and *2*) determine the impact, if any, of statins on those effects. New Zealand White rabbits receiving or not receiving atorvastatin were treated with dalcetrapib and anacetrapib for 2 weeks. We measured lipoprotein cholesterol profile biochemically and by fast protein liquid chromatography (FPLC), the apo E level by SDS-PAGE and its distribution among HDL subspecies by 2D-nondenaturing gradient gel electrophoresis (2D-NDGGE), and the ability of HDL-enriched (apo B-depleted) serum to compete for the association of human LDL with the LDLr in HepG2 cells. Samples of patients from dal-PLAQUE 2, a phase 2 carotid imaging trial of dalcetrapib, were also studied to evaluate lipoprotein concentrations by proton NMR spectroscopy and apo E level and distribution among HDL subspecies by ELISA and 2D-NDGGE, respectively.

## Materials and methods

### Materials

HepG2, THP-1, and RAW 264.7 cells were obtained from the ATCC (Manassas, VA). Anacetrapib was obtained from Acorn Pharmatech (Redwood City, CA) and dalcetrapib was produced and provided by Hoffmann-La Roche Ltd. (Basel, Switzerland).

### Animals and diet

**Fig. 1:**
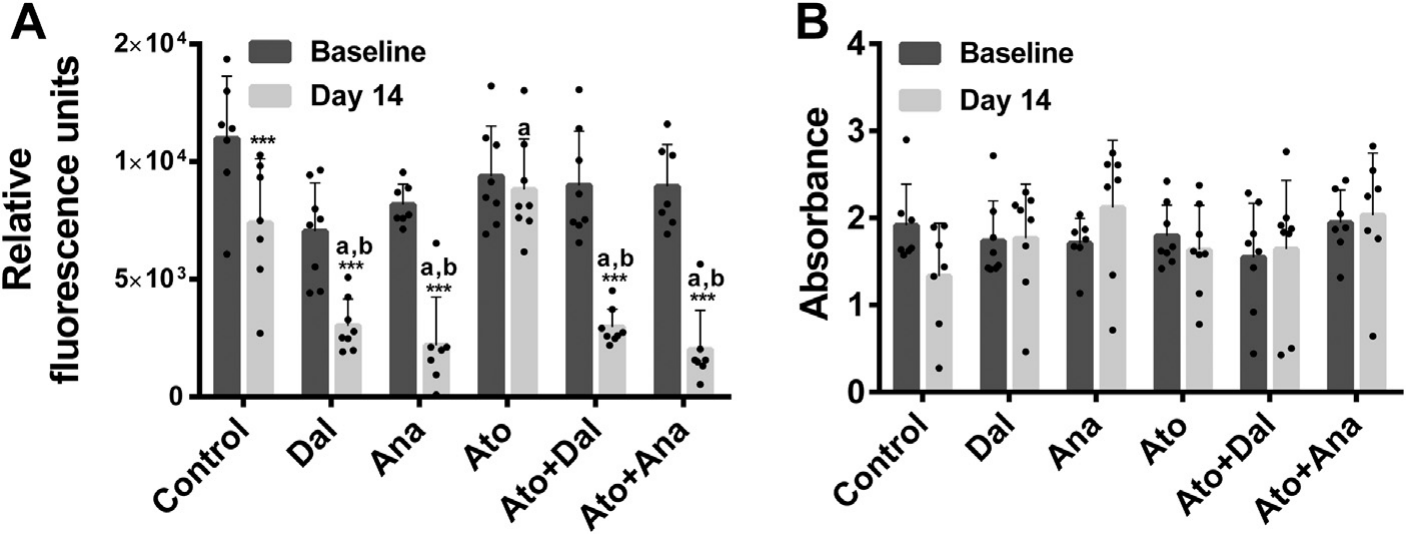
Effects of CETPi in the absence or presence of atorvastatin on rabbit plasma CETP activity and mass. CETP activity (A) and mass (B) were evaluated in rabbits treated with dalcetrapib or anacetrapib in the presence or absence of atorvastatin. Plasma at baseline and day 14 was used for measurement of CETP activity and mass. Results are presented as mean ± SD of 7–8 animals. ANCOVA analysis was performed. ^a^*P* < 0.05 compared to control group, ^b^*P* < 0.05 compared to atorvastatin group. Time comparisons within group were done using the analysis of least square means from the ANCOVA model. ∗∗∗*P* < 0.001 versus baseline. CETP, cholesteryl ester transfer protein; CETPi, CETP inhibitor.

**Fig. 2:**
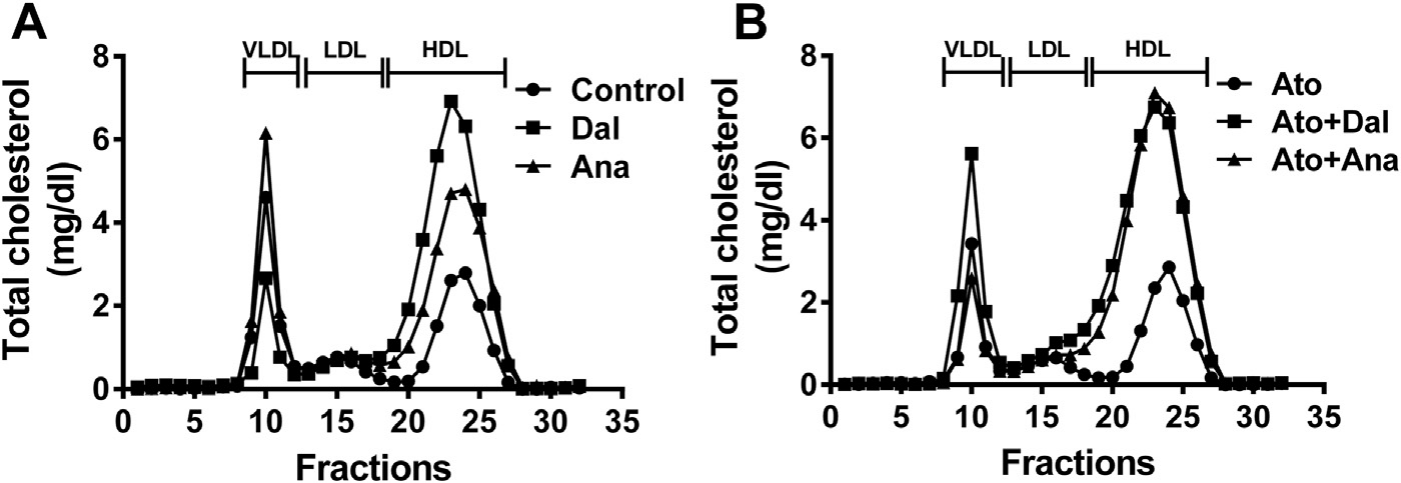
Effects of CETPi in the absence or presence of atorvastatin on rabbit FPLC lipid profile. Rabbits were treated with dalcetrapib or anacetrapib in the absence (A) or presence (B) of atorvastatin. At day 14, plasma lipoproteins from day 14 samples were separated by FPLC and total cholesterol concentrations were obtained in each fraction. Results are presented as mean of 7–8 animals. CETPi, cholesteryl ester transfer protein inhibitor; FPLC, fast protein liquid chromatography.

Male New Zealand White rabbits (3.0 kg, aged 12–13 weeks) were first acclimatized for 2 weeks under moderate caloric restriction (∼80% of ad libitum caloric intake) presented as 125 g (or 37.1 g/kg body weight) per day of cholesterol-free diet (32% energy from protein, 13% from fat, and 55% from carbohydrates) (Envigo, Madison, WI). Before starting the experimental diet, rabbits were randomized, according to their baseline HDL-C levels, to receive food as above to achieve doses of dalcetrapib of 300 mg/kg body weight (n = 8), or of anacetrapib of 30 mg/kg body weight (n = 7), and/or of atorvastatin of 2.5 mg/kg body weight (n = 8) or food only (n = 7) (supplemental Fig. S1). Food consumption and body weight were recorded throughout the studies to ensure adequate drug administration. Blood samples were obtained from an ear vein 1 day before treatment and on day 14 from animals fasted for ≥5 h. Plasma total cholesterol, HDL-C, LDL-C, and triglycerides (TG) levels were measured with an automated chemistry analyzer (Dimension RxL Max, Dade Behring, Deerfield, IL). All rabbit experiments were approved by the Animal Care and Use Committee of the Montreal Heart Institute. The results from control, dalcetrapib-, and anacetrapib-treated rabbits presented in Table 1, Fig. 1, Fig. 2 were previously published in the Journal of Lipid Research (28). They are used to provide a side-by-side comparison with animals treated with atorvastatin alone or in combination with a CETPi.

**Table 1:** Impact of CETPi and atorvastatin on New Zealand White rabbit lipid profiles

Group		Control		Dalcetrapib		Anacetrapib		Atorvastatin		Atorvastatin + Dalcetrapib		Atorvastatin + Anacetrapib	
Day		0	14	0	14	0	14	0	14	0	14	0	14
TC, mmol/l	Mean (SD)	0.58 (0.08)	0.57 (0.10)	0.58 (0.14)	1.06^a^^,^^b^^,^^e^ (0.42)	0.62 (0.12)	0.98^a^^,^^b^^,^^d^ (0.27)	0.64 (0.12)	0.48 (0.14)	0.61 (0.25)	1.32^a^^,^^b^^,^^e^ (0.71)	0.70 (0.17)	1.12^b^^,^^d^ (0.48)
HDL-C, mmol/l	Mean (SD)	0.32 (0.15)	0.21 (0.09)	0.38 (0.12)	0.69^a^^,^^b^^,^^d^ (0.34)	0.35 (0.12)	0.57^a^^,^^b^^,^^c^ (0.29)	0.38 (0.16)	0.18^c^ (0.09)	0.36 (0.27)	0.74^a^^,^^b^^,^^e^ (0.47)	0.47 (0.20)	0.78^a^^,^^b^^,^^d^ (0.46)
LDL-C, mmol/l	Mean (SD)	0.16 (0.07)	0.14 (0.06)	0.15 (0.04)	0.15^b^ (0.07)	0.15 (0.03)	0.17^b^ (0.09)	0.18 (0.09)	0.10^d^ (0.03)	0.15 (0.05)	0.21^a^^,^^b^^,^^c^ (0.15)	0.16 (0.04)	0.13 (0.08)
TG, mmol/l	Mean (SD)	1.08 (1.08)	1.08 (0.84)	0.61 (0.21)	0.76 (0.29)	0.79 (0.57)	1.47^b^^,^^d^ (1.54)	0.71 (0.47)	0.89 (0.42)	1.02 (1.03)	1.31 (1.97)	0.65 (0.22)	0.78 (0.52)

TC, total cholesterol; TG, triglycerides.

Rabbits were fed for 2 weeks with a diet containing dalcetrapib or anacetrapib in the absence or presence of atorvastatin, and lipid profiles were evaluated biochemically at baseline and at day 14. Results are shown as the mean ± SD of 7–8 animals. ANCOVA analysis was performed.

a *P* < 0.05 compared to control group.

b *P* < 0.05 compared to atorvastatin group. Time comparisons within group were done using the analysis of least square means from the ANCOVA model.

c *P* < 0.05.

d *P* < 0.01.

e *P* < 0.001 versus baseline.

### Plasma samples from dal-PLAQUE-2 trial

The dal-PLAQUE-2 study (NCT01059682) was a multi-center, randomized, double-blind, placebo-controlled phase 3 trial of dalcetrapib (600 mg daily) on carotid imaging changes, performed in accordance with Declaration of Helsinki guidelines. Inclusion criteria required that patients had stable coronary artery disease (CAD) as evidenced angiographically, ultrasound evidence of carotid artery disease, and treated appropriately for dyslipidemia (24, 25). Plasma (EDTA) samples obtained from venous blood of dal-PLAQUE-2 participants at baseline and after 12 months of treatment with dalcetrapib or placebo were provided by Hoffmann-La Roche (Basel, Switzerland). The research protocols were approved by the relevant institutional review boards or ethics committees, and all human participants gave written informed consent.

### CETP mass and activity

Measurement of CETP mass in plasma was performed by ELISA using mouse mAb clone 68/5 as capture antibody for rabbit CETP. The detection antibody used was JRC-2 (rabbit pAb) antibody conjugated to horseradish peroxidase as previously described (28). All these antibodies were kindly provided by F. Hoffmann-La Roche Ltd. Rabbit CETP activity was determined by an ex vivo CETP fluorescent activity kit (Roar Biomedical Inc., New York, NY) (29). Briefly, the assays were performed by incubating 95% (v/v) plasma, or assay buffer for blank, with the commercial reagent up to a total volume of 105 μl at 37°C for 90 min and fluorescence was read at 528 nm.

### Cholesterol distribution in lipoprotein classes by FPLC

Rabbit plasma was separated by FPLC-size-exclusion chromatography using a Superose-6 10/300 GL column, as described previously (30). The cholesterol content of each fraction was measured with a fluorometric assay (31).

### Lipoprotein particle concentrations by proton NMR spectroscopy analysis

Lipoprotein particle concentrations were measured by proton NMR spectroscopy analysis (LipoProfile, LipoScience, Raleigh, NC) on serum samples.

### Rabbit apo B-containing lipoproteins and HDL isolation

Lipoproteins were isolated from pools of EDTA-plasma from rabbits treated or not with CETPi and/or atorvastatin. Before isolation, 0.01% EDTA, 0.02% sodium azide, 10 μM PMSF, and 10 μM butylated hydroxytoluene were added to the plasma. The apo B-containing lipoproteins with a density of 1.006–1.055 g/ml and HDL particles with a density of 1.055–1.21 g/ml were prepared by ultracentrifugation as described previously (32).

### LC-MS/MS analysis

HDL was isolated by ultracentrifugation from pooled plasmas of rabbits treated or not with CETPi and/or atorvastatin. Then, apo E peptide counts were measured by LC-MS/MS analysis after tryptic digestion of HDL proteins and desalting of tryptic peptides (Caprion Biosciences Inc., Montreal, QC).

### Plasma concentration of apo E

Apo E concentration in rabbit and human plasma was measured by SDS-PAGE followed by Western blotting with anti-apo E (Meridian Life Science Inc., Memphis, TN) and by ELISA (Cell Biolabs Inc., San Diego, CA), respectively. The level of apo E in plasma depleted of apo B-containing lipoproteins, obtained after polyethylene glycol (PEG) precipitation was also determined as previously described (28).

### 2D-nondenaturing gradient gel electrophoresis

The 2D-NDGGE was performed by a modification of the protocol described by Asztalos *et al.* (33). Briefly, samples were electrophoresed on 0.75% agarose gel in Tris-glycine buffer (pH 8.6) at constant voltage (100 V). Then, agarose gel sections were placed onto a 4%–30% polyacrylamide nondenaturing gradient gel. The second dimension was run at 150 V for 24 h at 4°C in a buffer containing 90 mM Tris, 80 mM boric acid, and 2.5 mM EDTA (pH 8.3). After electrophoresis, lipoproteins were transferred to a 0.2 μm pore size nitrocellulose membrane (Bio-Rad, Mississauga, Ontario, Canada) at 12 V for 20 h at 4°C in a Tris-glycine buffer without methanol. After transfer, the membrane was blocked and immunodetection was performed with goat antibody to human apo E (Meridian Life Science Inc., Memphis, TN) followed by incubation with horseradish peroxidase-conjugated secondary antibody (Abcam, Toronto, ON, Canada) and by enhanced chemiluminescence detection on a Chemidoc MP Imaging System (Bio-Rad, Mississauga, Ontario, Canada). For fluorescence duplex Western blot, membrane immunoblotting was done with anti-apo E and goat antibody to human apo A-I (Meridian Life Science Inc., Memphis, TN) labeled with Alexa Fluor 647 or 546 (Thermo Fisher Scientific, Burlington, ON, Canada), respectively.

### Competition assays

HDL-enriched (apo B-depleted) rabbit serum was used as a competitor for the association of human DiI (Thermo Fisher Scientific, Burlington, ON, Canada)-labeled LDL on HepG2 cells treated for 16 h with 5 μM atorvastatin to induce LDLr levels. Briefly, DiI-LDL (7.5 μg/ml protein/ml), labeled as described by Pitas *et al.* (34), was incubated for 1 h 30 min at 37°C with cells treated in the presence or absence of 1% of apo B-depleted rabbit serum. At the end of the incubation, cell monolayers were washed once with PBS containing 0.2% BSA (PBS-BSA) and twice with PBS. Cells were solubilized in PBS plus 5 μM cholic acid and 0.1% Triton X-100. Solubilization buffer was then harvested, centrifuged for 5 min at 1,000 *g*, and transferred into a black plate for fluorescence measurement. The results are presented as the percentage of DiI-LDL association remaining in the presence of apo B-depleted serum relatively to the association obtained with DiI-LDL alone.

### Cellular cholesterol efflux capacity of serum

THP-1 monocytes were differentiated in macrophages with 100 ng/ml phorbol 12-myristate 13-acetate for 72 h. Then, cells were labeled in RPMI containing 2 μCi/ml [1,2-3H]-cholesterol plus 1% FBS in the presence of 50 μg/ml of human acetylated LDL for 24 h at 37°C. Then, cells were equilibrated with RPMI containing 0.2% BSA for 18 h at 37°C. An efflux assay was performed in the absence or presence of 3% rabbit apo B-depleted serum for 4 h. The apo B-depleted serum was obtained by adding 0.4 vol of PEG solution (20% PEG 6000 in 200 mM glycine buffer pH 7.4) to 1 vol of plasma. Samples were vortex-mixed, incubated for 20 min at 4°C, and centrifuged at 10,000 *g* for 30 min at 4°C. At the end of the incubation, the medium was harvested and cells were solubilized. Medium and cells were counted for radioactivity in a β-counter. The percentage of efflux was calculated by subtracting the radioactive counts in the medium in the absence of cholesterol acceptors from the radioactive counts in the presence of acceptor and then dividing by the sum of the radioactive counts in the medium plus the cell fraction.

### Quantification of mRNA expression by RT-quantitative PCR

The murine macrophage cell line RAW 264.7 was maintained in DMEM supplemented with 10% FBS at 37°C in 5% CO_2_ environment. The cells were preincubated for 30 min with or without apo B-depleted serum prior to addition of 25 ng/ml lipopolysaccharide (LPS). Total RNA was extracted using RNeasy isolation kits according to the manufacturer’s protocol. Complementary DNA was synthesized with components from High-Capacity complementary DNA RT kits and with the use of MultiScribe Reverse Transcriptase, according to the manufacturer’s procedures. Primers were designed using the Beacon designer software v.8 and sequences are in supplemental Table S1. The reference genes for normalization, SDHA and B2M, were selected by using the Bio-Rad CFX Maestro software which uses the GeNorm method. The quantitative PCR was performed with SYBR-Green reaction mix. The quantitative PCR conditions consisted of an initial denaturation at 95°C for 5 min, followed by 40 cycles of amplification, with each cycle consisting of 95°C for 15 s and 57°C for 60 s. Results were analyzed with the delta-delta Ct method done with the Bio-Rad CFX Maestro software.

### Statistical analysis

Statistical analyses were performed independently by the Montreal Health Innovations Coordinating Center statistical analysis group. Data are shown as mean ± SD unless stated otherwise. An ANCOVA model adjusting for baseline value was used for rabbit data to compare change from baseline to day 14 between groups. Time comparisons within group were done using the analysis of least square means from the ANCOVA model. One-way ANOVA was used to evaluate change in the lipid composition of lipoprotein particles isolated from New Zealand White rabbits. For longitudinal clinical data, ANCOVA analysis adjusting for baseline value was performed to determine if change from baseline to 1 year is different between groups. Paired *t*-tests were performed for time comparisons. One-way repeated measures ANOVA was performed to determine the effect of dalcetrapib on apo E concentration in plasma of stable CAD patients from the dal-PLAQUE-2 study. A two-tailed *P*-value <0.05 was considered statistically significant. No correction for multiple comparisons was done. All statistical analysis was performed using SAS version 9.4 (SAS Institute, NC) or R software.

## Results

### Effects of CETPi and atorvastatin on CETP activity and mass

CETP inhibition was evaluated at the end of the treatment by measuring CETP activity in plasma of CETPi-treated rabbits receiving or not receiving atorvastatin. As shown in Fig. 1A, the inhibition of CETP by dalcetrapib and anacetrapib was not affected by the presence of atorvastatin. Indeed, dalcetrapib reduced CETP activity by 57% (*P* < 0.001) and 67% (*P* < 0.001) in the absence or presence of atorvastatin, respectively (Fig. 1A). Similarly, CETP activity was reduced by 73% (*P* < 0.001) and 77% (*P* < 0.001) in animals receiving anacetrapib in the absence or presence of atorvastatin, respectively (Fig. 1A). This CETP inhibition caused by dalcetrapib and anacetrapib was significantly different when compared to the control and atorvastatin group (*P* < 0.05), although a reduction (−33%, *P* < 0.001) of CETP activity was also observed in control animals. We also measured the level of plasma CETP mass found in animals treated or not with CETPi and atorvastatin. CETP mass was not significantly modulated by any treatment (Fig. 1B).

### Effects of CETPi and atorvastatin on the lipid profile

We next evaluated the effect of CETPi in rabbits receiving or not receiving atorvastatin on plasma lipid levels. As shown in Table 1, although atorvastatin reduced the level of HDL-C (−53%, *P* < 0.05), the CETPi-driven increase of HDL-C was not affected. Indeed, dalcetrapib increased HDL-C by 1.8-fold (*P* < 0.01) and 2.1-fold (*P* < 0.001) in the absence or presence of atorvastatin, respectively. This increase of HDL-C caused by dalcetrapib was significantly different compared to the control and atorvastatin group (*P* < 0.05). Both CETPi administered to rabbits receiving or not receiving atorvastatin increased cholesterol in FPLC fractions corresponding to HDL (Fig. 2 and supplemental Fig. S2). Furthermore, more HDL was found in earlier fractions, indicating the generation of larger HDL. This result was supported by the increase of large HDL particle concentration following CETPi administration (supplemental Fig. S3). Atorvastatin reduced LDL-C by 44% (*P* < 0.01) and 29% (*P* = 0.14) compared to the baseline level and control group after 2 weeks, respectively (Table 1). A reduction of 23% in LDL particle concentration was observed in atorvastatin-treated rabbits compared to control animals, a difference which was not statistically significant (supplemental Fig. S3). While both CETPi did not significantly affect LDL-C in the absence of atorvastatin, LDL-C was increased by 40% with dalcetrapib (*P* < 0.05) but not anacetrapib when coadministered with atorvastatin (Table 1). This increase caused by dalcetrapib was significantly different compared to the atorvastatin group (*P* < 0.05). Nonsignificant increases of plasma LDL particle concentration were observed with CETPi (supplemental Fig. S3).

### Effects of CETPi and atorvastatin on the lipid composition of HDL and apo B-containing lipoproteins isolated from rabbits

To assess whether the higher HDL-C level induced by CETPi results in an increase of HDL size, we evaluated the cholesterol/protein ratio of HDL particles isolated from rabbits treated with CETPi and atorvastatin. This ratio was not affected by atorvastatin (Table 2). The increase of cholesterol/protein ratio in HDL from rabbits treated with dalcetrapib increased by 92% (*P* < 0.001) and 85% (*P* < 0.001) in the absence or presence of atorvastatin, respectively. The corresponding increases with anacetrapib were 97% (*P* < 0.001) and 59% (*P* < 0.001). Both CE and free cholesterol associated to HDL were increased by dalcetrapib and anacetrapib, and this effect was not affected by atorvastatin. Dalcetrapib and anacetrapib, in the absence or presence of atorvastatin, significantly reduced TG found in HDL, indicating that TG transfer from apo B-containing lipoproteins was inhibited.

**Table 2:** Impact of CETPi and atorvastatin administration on lipid composition of HDL particles isolated from New Zealand White rabbits

Ratio	Control	Dalcetrapib	Anacetrapib	Atorvastatin	Atorvastatin + Dalcetrapib	Atorvastatin + Anacetrapib
Chol/Prot	0.182 ± 0.003	0.337 ± 0.012^a^^,^^b^	0.290 ± 0.005^a^^,^^b^	0.180 ± 0.005	0.345 ± 0.013^a^^,^^b^	0.354 ± 0.016^a^^,^^b^
CE/Prot	0.219 ± 0.011	0.440 ± 0.013^a^^,^^b^	0.381 ± 0.006^a^^,^^b^	0.224 ± 0.007	0.437 ± 0.025^a^^,^^b^	0.470 ± 0.021^a^^,^^b^
FC/Prot	0.052 ± 0.005	0.076 ± 0.009^a^^,^^b^	0.064 ± 0.009^b^	0.046 ± 0.009	0.085 ± 0.009^a^^,^^b^	0.074 ± 0.009^a^^,^^b^
TG/Prot	0.171 ± 0.023	0.060 ± 0.008^a^^,^^b^	0.083 ± 0.012^a^^,^^b^	0.225 ± 0.031	0.073 ± 0.009^a^^,^^b^	0.065 ± 0.009^a^^,^^b^

CE, cholesterol ester; Chol, cholesterol; FC, free cholesterol; TG, triglycerides.

Rabbits were fed for 2 weeks with a diet containing dalcetrapib or anacetrapib in the absence or presence of atorvastatin, and lipid composition of HDL isolated by ultracentrifugation was evaluated biochemically. One-way ANOVA was used for statistical analysis.

a *P* < 0.05 compared to control group.

b *P* < 0.05 compared to atorvastatin group.

The impact of CETPi on HDL size was evaluated by detecting apo A-I in rabbit-purified HDL separated by one-dimensional-NDGGE followed by quantification of HDL subclasses’ percentages in each preparation (supplemental Fig. S4). Percentages of large HDL (8.8 nm–12.2 nm) were increased by dalcetrapib (+31% and +20%) and by anacetrapib (+11% and +15%) compared to HDL from control and atorvastatin groups, respectively (supplemental Fig. S4). The impact of CETPi on HDL size was also assessed by measuring apo A-I distribution in HDL subclasses following rabbit plasma separation by one-dimensional-NDGGE. The apo A-I associated with total HDL was increased by dalcetrapib (+106%, *P* < 0.05; +151%, *P* < 0.05) and anacetrapib (+81%, *P* < 0.05; +57%, *P* < 0.05) compared to control and atorvastatin groups and this increase was found to be associated with both large and small migrating HDL (supplemental Fig. S5). Thus, the increased HDL cholesterol/protein ratio, percent large HDL particles in isolated lipoproteins, and apo A-I level associated with large HDL induced by dalcetrapib and anacetrapib are in line with the generation of larger HDL particles.

We also isolated apo B-containing lipoproteins and analyzed their composition to evaluate the impact of CETP inhibition. Atorvastatin reduced the cholesterol/protein ratio by 18% (*P* < 0.001) in apo B-containing lipoproteins compared to control (Table 3). This cholesterol/protein ratio in apo B-containing lipoproteins isolated from rabbits treated with dalcetrapib only or in combination with atorvastatin increased by 26% (*P* < 0.001) and 59% (*P* < 0.001), respectively (Table 3). Anacetrapib increased this ratio in apo B-lipoproteins in the absence (+13%, *P* < 0.001) or presence (+15%, *P* < 0.001) of atorvastatin. The aforementioned effects induced by CETPi and atorvastatin were found for both the ratios of CE and free cholesterol to protein, while TG was not modulated by any treatment (Table 3).

**Table 3:** Impact of CETPi and atorvastatin administration on lipid composition of apo B-containing lipoproteins isolated from New Zealand White rabbits

Ratio	Control	Dalcetrapib	Anacetrapib	Atorvastatin	Atorvastatin + Dalcetrapib	Atorvastatin + Anacetrapib
Chol/Prot	0.493 ± 0.018	0.622 ± 0.007^a^^,^^b^	0.558 ± 0.030^a^^,^^b^	0.403 ± 0.008^a^	0.641 ± 0.008^a^^,^^b^	0.463 ± 0.015^a^^,^^b^
CE/Prot	0.427 ± 0.031	0.524 ± 0.043^a^^,^^b^	0.417 ± 0.025	0.287 ± 0.034^a^	0.485 ± 0.028^b^	0.314 ± 0.066^a^
FC/Prot	0.239 ± 0.034	0.310 ± 0.033^a^^,^^b^	0.310 ± 0.040^a^^,^^b^	0.233 ± 0.028	0.353 ± 0.021^a^^,^^b^	0.277 ± 0.038
TG/Prot	2.107 ± 0.831	2.003 ± 0.783	2.021 ± 0.814	2.532 ± 1.032	2.073 ± 0.830	1.993 ± 0.783

CE, cholesterol ester; Chol, cholesterol; FC, free cholesterol; TG, triglycerides.

Rabbits were fed for 2 weeks with a diet containing dalcetrapib or anacetrapib in the absence or presence of atorvastatin, and lipid composition of apoB-containing lipoproteins isolated by ultracentrifugation was evaluated biochemically. One-way ANOVA was used for statistical analysis.

a *P* < 0.05 compared to control group.

b *P* < 0.05 compared to atorvastatin group.

### Effects of CETPi and atorvastatin on the concentration and distribution of apo E in lipoproteins and HDL subclasses

**Fig. 3:**
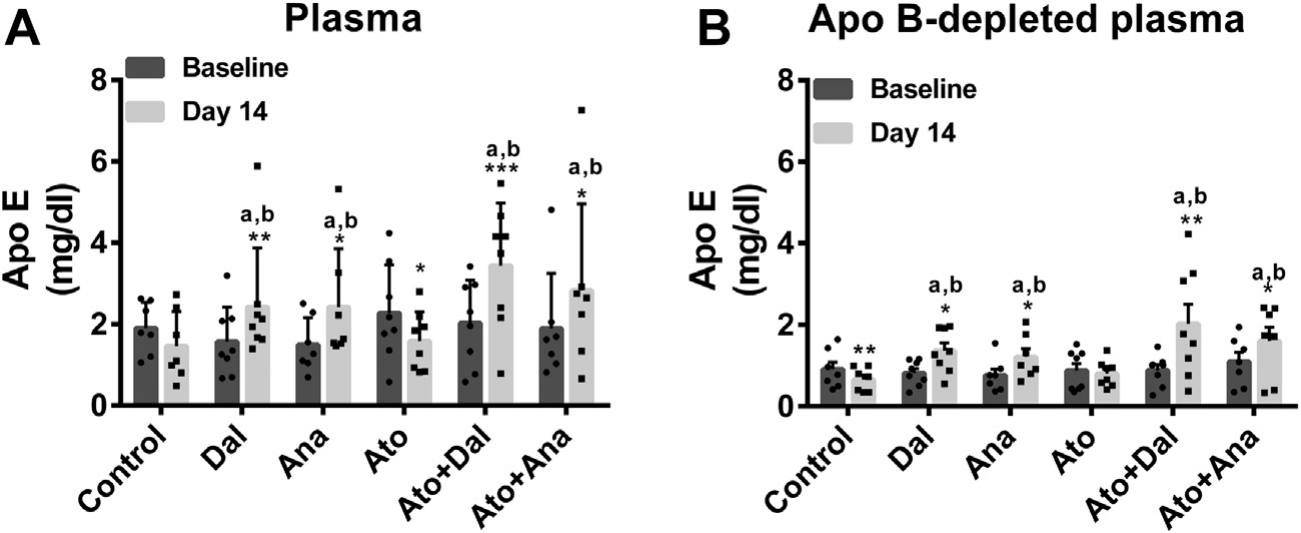
Effects of CETPi in the absence or presence of atorvastatin on the plasma level of apoE. Rabbits were treated with dalcetrapib or anacetrapib in the presence or absence of atorvastatin. Plasmas from baseline and day 14 were depleted or not of the apo B-lipoproteins and used for SDS-PAGE electrophoresis. Densitometric analysis of apo E Western blots for complete (A) and apo B-containing lipoproteins depleted plasma (B). Results are presented as mean ± SD of 7–8 animals. ANCOVA analysis was performed. ^a^*P* < 0.05 compared to control group, ^b^*P* < 0.05 compared to atorvastatin group. Time comparisons within group were done using the analysis of least square means from the ANCOVA model. ∗*P* < 0.05, ∗∗*P* < 0.01, ∗∗∗*P* < 0.001 versus baseline. apo E, apolipoprotein E; CETPi, cholesteryl ester transfer protein inhibitor.

To characterize the impact of CETPi and atorvastatin on the apolipoprotein composition of large HDL, we evaluated the concentration of apo E in total and apo B-depleted plasma by using a known concentration of purified commercial apo E as standard. The plasma concentration of apo E was increased by 54% (*P* < 0.01) and 69% (*P* < 0.001) in rabbits receiving dalcetrapib alone or in combination with atorvastatin (Fig. 3A). Apo E was increased with anacetrapib by 62% (*P* < 0.05) and 49% (*P* < 0.005) in the absence or presence of atorvastatin (Fig. 3A). This increase caused by dalcetrapib and anacetrapib was significantly different compared to the control and atorvastatin group (*P* < 0.05). To determine whether this higher level of apo E was associated with HDL or apo B-containing lipoproteins, we measured by Western blotting the level of apo E found in apo B-depleted plasma. Apo E was increased by 67% (*P* < 0.05) and 127% (*P* < 0.01) in the apo B-depleted plasma of rabbits receiving dalcetrapib alone or in combination with atorvastatin, respectively (Fig. 3B). Anacetrapib increased the level of apo E found in apo B-depleted plasma by 57% (*P* < 0.05) and 48% (*P* < 0.05) when administered in the absence or presence or atorvastatin, respectively (Fig. 3B). These effects support the generation of large HDL particles containing apo E by CETPi, since over 50% of the increase of apo E was associated with apo B-depleted plasma. The increase caused by dalcetrapib and anacetrapib in apo B-depleted plasma was significantly different compared to the control and atorvastatin groups (*P* < 0.05). Proteomic analysis of isolated HDL showed that apo E spectral counts were increased by dalcetrapib and anacetrapib (+67% and +18%, respectively) in the absence of atorvastatin (supplemental Table S2). Apo E peptides were also increased by 94% and 111% in HDL of rabbits receiving dalcetrapib or anacetrapib in combination with atorvastatin, although atorvastatin alone reduced the apo E level by 24% (supplemental Table S2). Proteomic results showed that apolipoprotein C-I and C-III peptide counts were increased similarly to apo E.

**Fig. 4:**
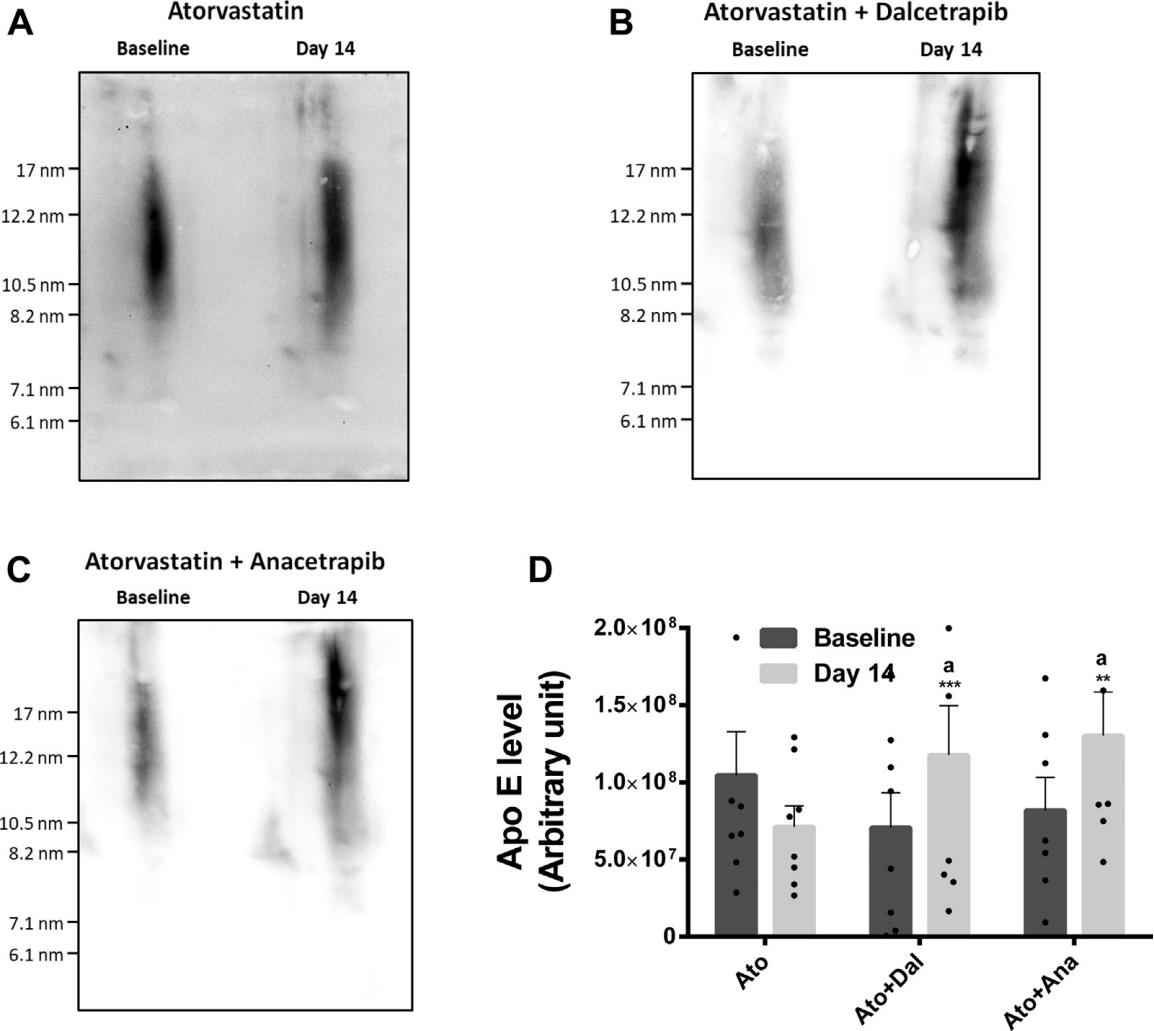
Effects of CETPi in the absence or presence of atorvastatin on rabbit apoE distribution and concentration in HDL subclasses. Rabbits were treated with dalcetrapib or anacetrapib in the presence of atorvastatin. Plasmas from day 14 were used for 4%-30% two-dimensional nondenaturating gradient gel electrophoresis (2D-NDGGE). Representative Western blots of plasma apoE of rabbits treated with (A) atorvastatin alone or in combination with (B) dalcetrapib or (C) anacetrapib. (D) Densitometric quantification of apoE Western blots presented as mean ± SD of 7 to 8 animals. ANCOVA was performed. ^a^*P*<0.05 compared to atorvastatin group. Time comparisons within group were done using the analysis of least square means from the ANCOVA model. ∗∗*P* < 0.01, ∗∗∗*P* < 0.001 versus baseline. CETPi, cholesteryl ester transfer protein inhibitor; apo E, apolipoprotein E.

Next, we established the distribution of rabbit apo E in HDL subclasses by 2D-NDGGE. As similar results for apo E concentrations were obtained in the presence or absence of atorvastatin, only rabbits treated with atorvastatin alone or combined with CETPi were analyzed. Apo E was found almost exclusively in large α-migrating HDL with a diameter above 8.2 nm (Fig. 4). While atorvastatin treatment had no impact on this parameter, addition of dalcetrapib or anacetrapib to atorvastatin treatment increased the apo E associated with large HDL particles by 66% (*P* < 0.001) and 59% (*P* < 0.01), respectively (Fig. 4). This increase caused by CETPi was significantly different when compared to atorvastatin (*P* < 0.05), which reduced the apo E associated with HDL by 32% (*P* = 0.10).

### Effects of CETPi and atorvastatin on interaction of apo E-containing HDL with hepatic LDLr

**Fig. 5:**
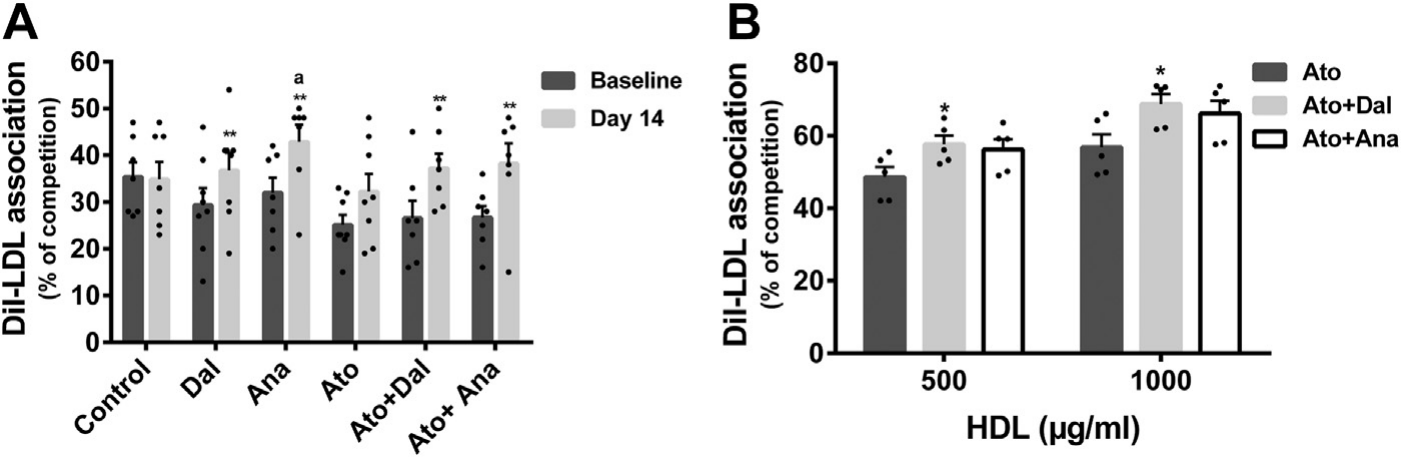
Effects of CETPi on the capacity of rabbit sera (A) or purified rabbit HDL (B) to compete for LDL association to HepG2 cells. Rabbits were treated with dalcetrapib or anacetrapib in the absence or presence of atorvastatin and plasmas were harvested at baseline and at day 14. A: The apo B-depleted lipoproteins (baseline and day 14) or (B) HDL isolated from plasma harvested at day 14 were used as competitors for the association of DiI-LDL to HepG2 cells. Results are presented as the percentage of DiI-LDL association competed in the presence of apo B-depleted serum and calculated from the association in the presence of apo B-depleted serum and the association in the absence of competitors. For competition with apo B-depleted plasma, results are presented as mean ± SEM of 7–8 animals. ANCOVA analysis was performed. ^a^*P* < 0.05 compared to control group. A time comparison within group was done using the analysis of least square means from the ANCOVA model. ∗∗*P* < 0.01 versus baseline. For competition with isolated HDL, results are presented as mean ± SEM of five assays. One-way ANOVA was used for statistical analysis. ∗*P* < 0.05 compared to atorvastatin group. CETPi, cholesteryl ester transfer protein inhibitor.

**Fig. 6:**
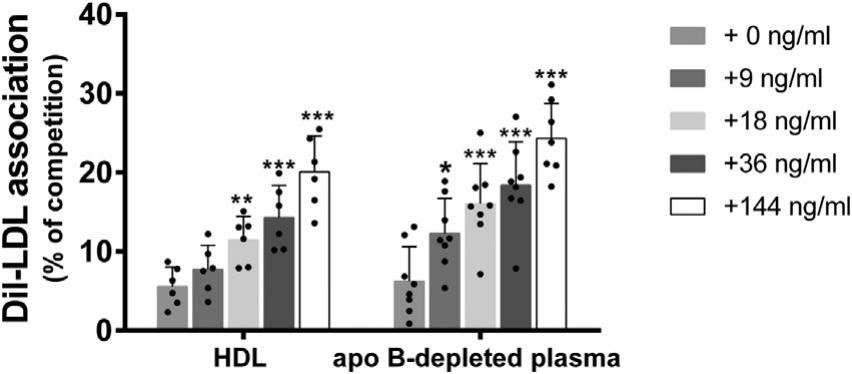
Effect of apo E on the capacity of human sera or purified human HDL to compete for LDL association to HepG2 cells. Human exogenous-purified apo E was incubated for 30 min with purified human HDL (50 μg/ml) or apo-B depleted serum (1%) before competition with DiI-LDL. Results are presented as the percentage of DiI-LDL association competed in the presence of apo B-depleted serum or isolated HDL and calculated from the association in the presence of apo B-depleted serum and the association in the absence of competitors. Results are presented as mean ± SD of 6–8 assays. One-way ANOVA was used for statistical analysis. ∗*P* < 0.05, ∗∗*P* < 0.01, ∗∗∗*P* < 0.001 compared to control. apo E, apolipoprotein E.

The ability of apo E-containing HDL to interact with LDLr was evaluated by determining the level of cell association of DiI-LDL in the presence of HDL-enriched (apo B-depleted) rabbit serum. Apo B-depleted serum of rabbits treated with dalcetrapib or anacetrapib for 14 days significantly increased the competition of LDL to HepG2 cells by 28% (*P* < 0.01) and 34% (*P* < 0.01) compared to baseline sera in the absence of atorvastatin (Fig. 5A). In the presence of atorvastatin, apo B-depleted serum of animals treated concomitantly with dalcetrapib or anacetrapib significantly increased the competition of LDL by 37% (*P* < 0.01) and 41% (*P* < 0.01). No significant difference was observed from baseline to day 14 between the control and atorvastatin groups (Fig. 5A). The competition assays were also conducted with plasma HDL isolated from rabbits treated with atorvastatin in the presence or absence of CETPi. HDL (500 μg/ml) isolated from rabbits treated with atorvastatin in the presence of dalcetrapib or anacetrapib significantly increased the competition with LDL to HepG2 cells by +19% (*P* < 0.05) and +16% (*P* = 0.07) compared to HDL from the atorvastatin alone group (Fig. 5B). Similar results were obtained with HDL used as competitors at 1,000 μg/ml (Fig. 5B). This increase of competition with LDL is associated with an increase of apo E detected by Western blot in HDL isolated from the dalcetrapib (+78%) and anacetrapib groups (+68%) (compared to the atorvastatin alone group, data not shown). Overall, these results suggest that large apo E-containing HDL has the capacity to associate with the LDLr. To support these competition results, we incubated exogenous purified human apo E with human HDL or human apo-B depleted plasma and used them for competition assays. Incorporation of exogenous apo E in human HDL (50 μg/ml) increased the competition of DiI-LDL association by HDL in a dose-dependent fashion (Fig. 6). Similar results were obtained when apo B-depleted plasma was used as competitor, indicating that enrichment of HDL with apo E increases the ability of HDL to compete LDL for cellular association.

### Effects of CETPi on atheroprotective properties of HDL

**Fig 7:**
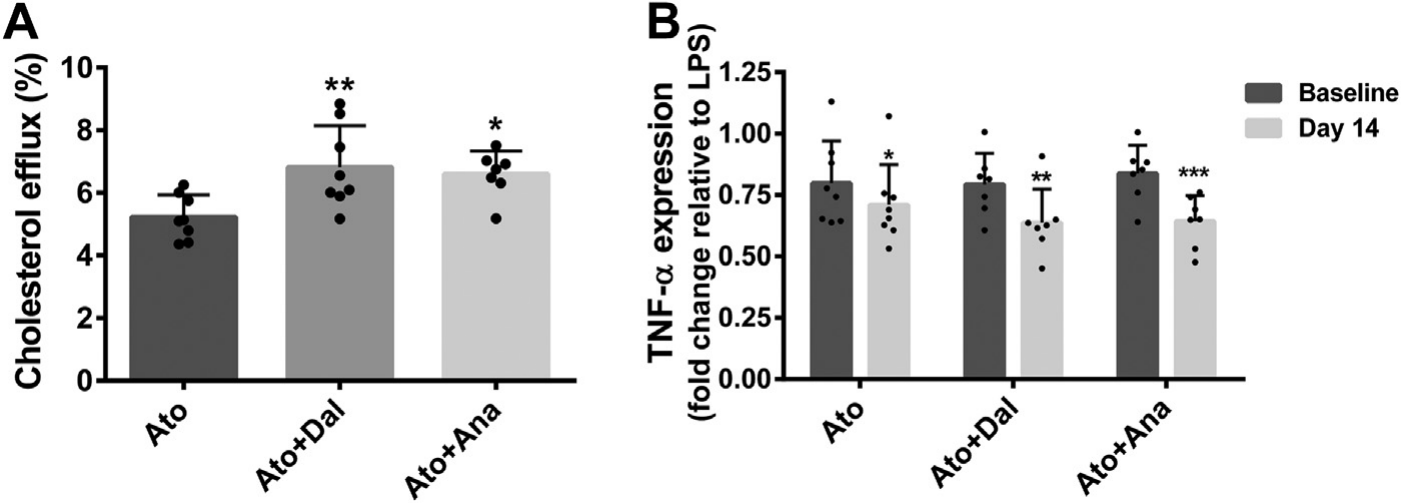
Impact of CETPi on atheroprotective functions of HDL. Rabbits were treated with atorvastatin in the presence or absence of dalcetrapib or anacetrapib and (A) apo B-depleted serum (3%) harvested at day 14 was used for cholesterol efflux assays with AcLDL-loaded THP-1 macrophages. Results are presented as mean ± SD of 7–8 animals. One-way ANOVA was performed. ∗*P* < 0.05, ∗∗*P* < 0.01 versus atorvastatin. B: RAW 264.7 macrophages were pretreated for 30 min with apo B-depleted serum (1%) harvested at day 0 and day 14 and mRNA modulation of TNF-α was measured following treatment of cells with LPS (25 ng/ml). Results are presented as mean ± SD of 7–8 animals. Time comparisons within group were done using the analysis of least square means from the ANCOVA model. ∗*P* < 0.05, ∗∗*P* < 0.01, ∗∗∗*P* < 0.001 compared to baseline. AcLDL, acetylated low density lipoprotein; CETPi, cholesteryl ester transfer protein inhibitor; LPS, lipopolysaccharide.

To assess whether the modulation of apo E level by CETPi had an impact on cholesterol efflux induced by HDL, we tested the capacity of apo B-depleted serum to accept cholesterol from acetylated LDL-loaded THP-1 macrophages. Figure 7A shows that cholesterol efflux induced by apo B-depleted serum from rabbits treated with atorvastatin in the presence of dalcetrapib or anacetrapib is increased by +31% (*P* < 0.01) and +26% (*P* < 0.05) compared to atorvastatin alone. The impact of apo E modulation on inflammation was evaluated by measuring the ability of apo B-depleted serum to prevent the mRNA upregulation of TNF-α induced by LPS in RAW 264.7 macrophages (Fig. 7B). While apo B-depleted serum from rabbits treated with atorvastatin decreased TNF-α expression by 11% (*P* < 0.05) compared to baseline, this reduction was enhanced by dalcetrapib and anacetrapib given that the mRNA level of TNF-α was reduced by 19% (*P* < 0.01) and by 24% (*P* < 0.001) compared to baseline, respectively.

### Effects of dalcetrapib on lipoprotein particle concentrations in patients with CAD

To determine if dalcetrapib has similar effects in humans, we first measured the lipoprotein particle concentrations in a subset of patients from the dal-PLAQUE-2 trial for which plasma was available. Dalcetrapib significantly increased the total concentration of HDL particles by 5.3% (*P* < 0.001, Table 4). This resulted from an increase of 68.7% (*P* < 0.001) in large HDL particles and a decrease in the most abundant small HDL particles (−9.1%, *P* < 0.001), while medium HDL particle concentration remained stable. These effects of dalcetrapib on HDL sizes were significantly different compared to placebo group (*P* < 0.05), since large HDL particles was increased by only 9.5% (*P* < 0.01) in placebo. Although intermediate-density lipoprotein and large LDL particles were increased by 16.1% and 37.5% (*P* < 0.001), dalcetrapib decreased the total LDL particle concentration by 3.1% (*P* < 0.05). This lower level of LDL particles was caused by a reduction of 19.7% (*P* < 0.001) in the concentration of small LDL, the more abundant LDL subclass in circulation. The modulation of LDL particles by dalcetrapib was significantly different from those measured in placebo group in which only large LDL particles concentration was slightly increased (11.2%, *P* < 0.05). Thus, dalcetrapib increases the concentration of large HDL particles in patients with CAD. Whether these large HDL particles were also enriched in apo E in such patients remained to be determined.

**Table 4:** Effect of dalcetrapib or placebo on lipoprotein concentrations measured by NMR in patients of the dal-PLAQUE-2 study

NMR LipoProfile (nmol/l)	Dalcetrapib n = 179		Placebo n = 178	
	Baseline	1 Year	Baseline	1 Year
Total HDL concentration	32.48 ± 5.92	34.20 ± 5.83^a^^,^^d^	32.68 ± 5.57	33.36 ± 5.64^b^
Large HDL	4.38 ± 2.68	7.39 ± 4.24^a^^,^^d^	4.22 ± 2.40	4.62 ± 2.90^c^
Medium HDL	9.34 ± 5.63	9.77 ± 5.10	9.40 ± 5.70	9.59 ± 5.17
Small HDL	18.75 ± 4.66	17.04 ± 5.99^a^^,^^d^	19.06 ± 4.63	19.14 ± 5.01
Total LDL concentration	822.32 ± 238.18	796.83 ± 293.91^a^^,^^b^	860.12 ± 248.53	878.32 ± 260.12
Intermediate-density lipoprotein	62.89 ± 46.23	73.00 ± 50.17	65.35 ± 61.45	70.22 ± 62.03
Large LDL	199.36 ± 140.93	274.05 ± 171.53^a^^,^^d^	199.11 ± 141.94	221.33 ± 156.13^b^
Small LDL	560.07 ± 220.65	449.78 ± 273.68^a^^,^^d^	595.66 ± 200.42	586.78 ± 242.72

Lipoprotein particle concentrations (nmol/l) in plasma were measured by NMR LipoProfile in patients enrolled in the dal-PLAQUE-2 substudy at baseline and 1 year of treatment in the placebo and dalcetrapib groups. ANCOVA analysis adjusting for baseline value was performed to determine if change at 1 year is different between groups.

a *P* < 0.05 compared to placebo group. Paired t-tests were performed for time comparisons.

b *P* < 0.05.

c *P* < 0.01.

d *P* < 0.001 versus baseline.

### Effects of dalcetrapib on the level and distribution of apo E in lipoproteins of CAD patients

**Fig. 8:**
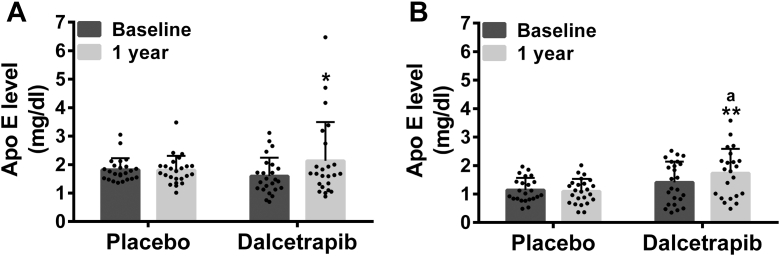
Effect of dalcetrapib on apoE concentration in humans. Plasma of stable CAD patients from the dal-PLAQUE-2 study was collected at baseline and after 1 year of dalcetrapib treatment (600 mg daily) or placebo. Then, plasmas were used to evaluate by ELISA the apo E concentration in (A) complete or in (B) apo B-containing lipoprotein-depleted plasmas. Results are presented as mean ± SD of 22–24 patients. ANCOVA analysis was performed. ^a^*P* < 0.001 compared to placebo. Time comparisons within group were done using the analysis of least square means from the ANCOVA model. ∗*P* < 0.05, ∗∗*P* < 0.01 versus baseline. apo E, apolipoprotein E; CAD, coronary artery disease.

To characterize the impact of dalcetrapib on the apolipoprotein composition of HDL, we first evaluated the level of apo E in plasma of patients treated or not treated with dalcetrapib for 1 year, by ELISA. As shown in Fig. 8, the concentration of apo E was increased in total plasma (33%, *P* < 0.05) and in apo B-depleted plasma (24%, *P* < 0.001) of patients treated with dalcetrapib compared with baseline values. Apo E concentration remained unchanged over time in the placebo group (Fig. 8).

**Fig. 9:**
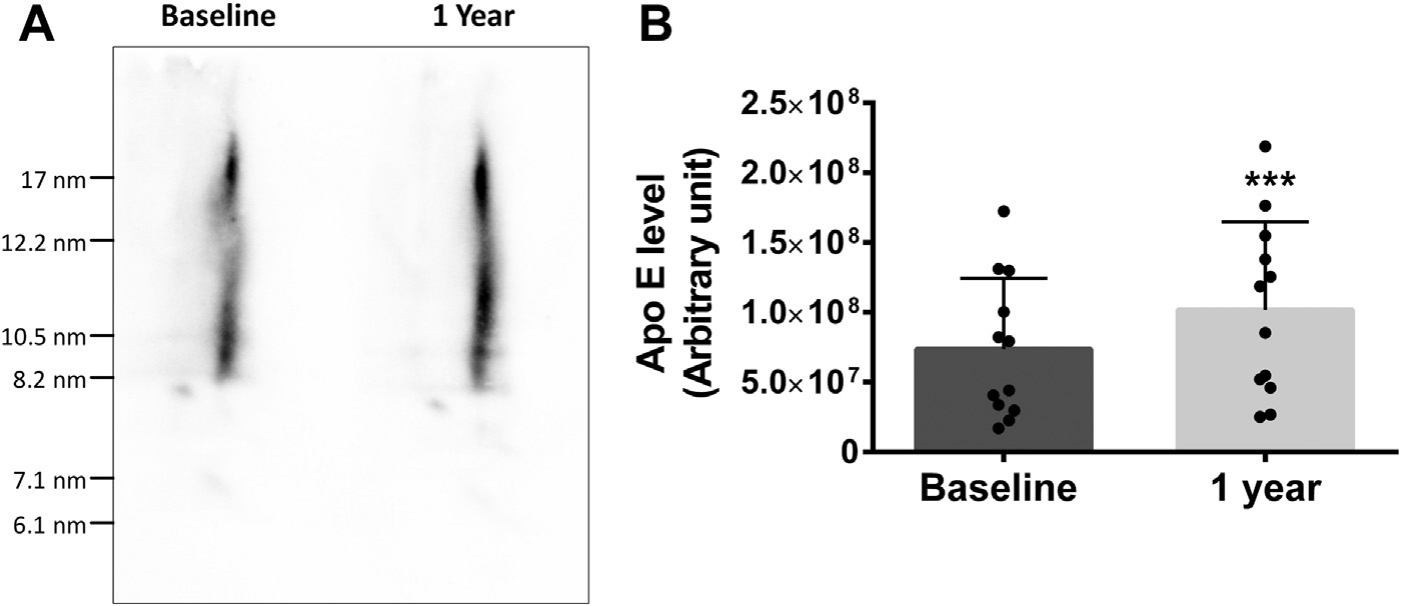
Effect of dalcetrapib on apo E concentration in human HDL subclasses. Plasma of stable CAD patients from the dal-PLAQUE-2 study was collected at baseline and after 1 year of dalcetrapib (600 mg daily) treatment. Then, plasmas were used for 4%–30% two-dimensional nondenaturating gradient gel electrophoresis (2D-NDGGE). A: Representative Western blots of plasma apo E in one patient at baseline and after 1 year of treatment with dalcetrapib. B: Densitometric analysis of apo E Western blots presenting the mean band intensity for each time point. Results are presented as mean ± SD of 12 subjects. One-way repeated measures ANOVA was performed. ∗∗∗*P* < 0.001 versus baseline. CAD, coronary artery disease.

We also characterized the distribution of apo E in HDL subclasses by 2D-NDGGE. Figure 9A shows that apo E was associated with large α-migrating HDL with a diameter >8.2 nm. In agreement with ELISA results, dalcetrapib increased apo E associated with HDL by 47% (*P* < 0.001, Fig. 9B).

**Fig. 10:**
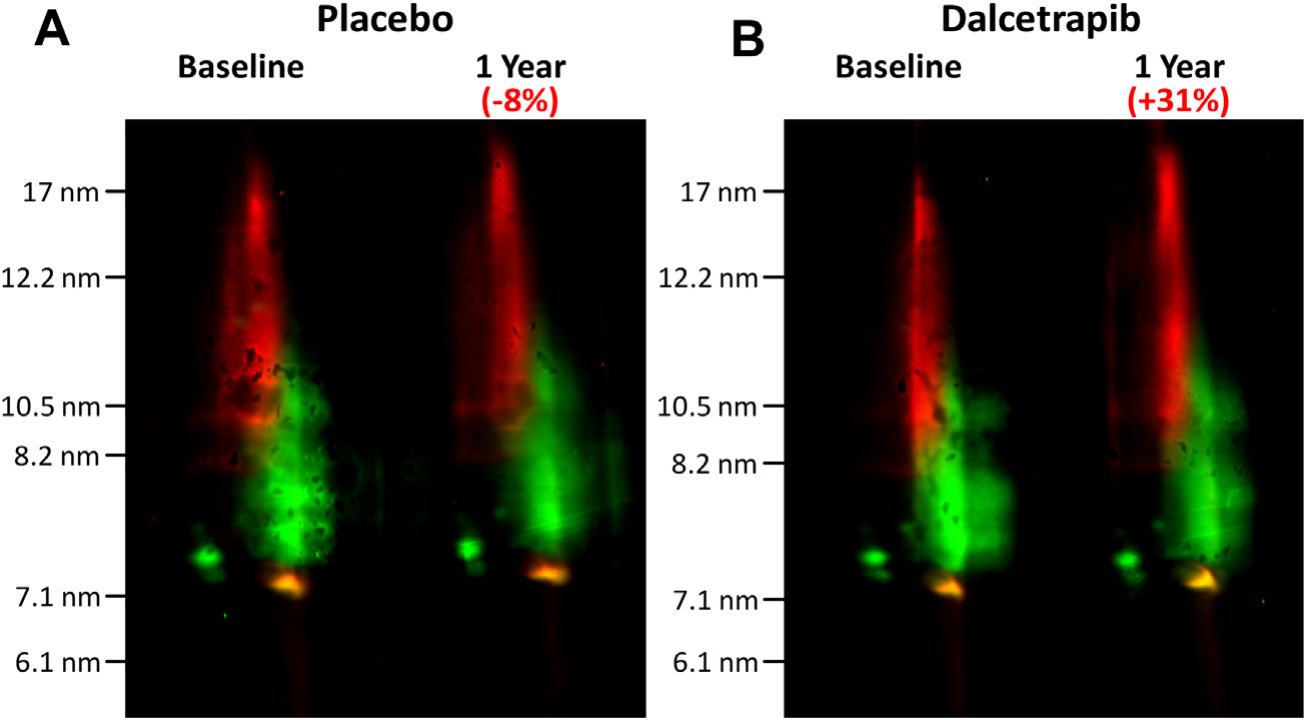
Effect of dalcetrapib on the distribution of apo A-I and apo E in human HDL subclasses. Plasma of stable CAD patients from the dal-PLAQUE-2 study was collected at baseline and after 1 year of dalcetrapib (600 mg daily) treatment or placebo. Then, 12 plasmas of the (A) placebo or (B) dalcetrapib group were pooled and used for 4%–30% two-dimensional nondenaturating gradient gel electrophoresis (2D-NDGGE). Western blots of apo A-I (green) and apo E (red) were done simultaneously with an anti-apo A-I and anti-apo E labeled with Alexa-Fluor-546 or -647, respectively. Densitometric analysis of apo E Western blots is indicated at the top of the gel as the relative change from baseline. apo A-I, apolipoprotein A-I; apo E, apolipoprotein E; CAD, coronary artery disease.

We also evaluated more precisely the apolipoprotein composition of these large HDL particles generated by dalcetrapib by fluorescent duplex Western blot. We searched simultaneously for apo A-I and apo E with specific Alexa-conjugated primary antibodies. Large α-migrating HDL particles containing apo E (shown in red) did not overlap with the apo A-I-containing HDL (shown in green) which were found predominantly in particles below 10 nm in diameter (Fig. 10A). The increase of 31% in apo E induced by dalcetrapib was only found in large HDL particles and not in smaller apo A-I-containing HDL (Fig. 10B). No modulation of apo E in any HDL subclass was observed in patients of the placebo group (Fig. 10A).

## Discussion

The positive results obtained in the REVEAL phase 3 clinical trial of anacetrapib (23) and the pharmacogenetics-guided dal-GenE dalcetrapib trial (24, 25, 26, 27) highlight the importance of better characterizing and understanding the effects of these CETPi on HDL particle composition and function. HDL particles play a central role in RCT by promoting cholesterol efflux and by being involved in the delivery of cholesterol to the liver (35). This last step is achieved by the direct and indirect pathways. The former involves the selective transfer of cholesterol from HDL to the liver via direct interactions with hepatic receptors such as SR-B1 (35). The indirect route consists of CETP-mediated transfer of CE from mature HDL to apo B-containing lipoproteins including LDL and VLDL, which will in turn be cleared by hepatic LDLr. Since CETPi can modulate the final steps of RCT, characterization of mature HDL particles modified by dalcetrapib and anacetrapib is warranted to evaluate their ability to deliver their cholesterol load to the liver. In the current study, we demonstrated that dalcetrapib and anacetrapib increase the concentration of apo E-containing HDL particles that have the capacity to associate with the hepatocyte’s LDLr.

Previous studies have demonstrated that CETP-deficient patients have a marked increase in plasma apo E (36, 37), suggesting that pharmacological inhibition of CETP activity would increase circulating levels of apo E. As expected, we showed using gel electrophoresis of rabbit plasmas and ELISA on human samples that total plasma apo E was increased by the administration of CETPi in both species. These results confirmed those previously obtained in rabbits treated with dalcetrapib (6) and in patients receiving torcetrapib (38), anacetrapib (11), and dalcetrapib (10, 39). This increase may be due to a higher production rate as indicated by such previous results with anacetrapib, without significant change in the clearance rate of apo E (11). However, additional studies were required to determine whether dalcetrapib also enhances the production rate of apo E and if apo E gene expression in liver (or other tissues) is induced by a direct action of CETPi or indirectly by an increase of cholesterol level in liver due to enhanced RCT.

As demonstrated by Western blotting on apo B-depleted plasma, we found that CETPi increases the level of apo E associated with HDL. We also found, by measuring the level of apo E in complete and apo B-depleted plasma by ELISA, that 60% of the apo E increase induced by dalcetrapib is associated with HDL in humans. It was previously demonstrated that patients with CETP deficiency (40, 41, 42) or those receiving dalcetrapib (10) have an increased level of apo E associated with HDL. However, the distribution of apo E among HDL particles following CETP inhibition remained unknown. Thus, to characterize the apolipoprotein composition of HDL caused by dalcetrapib administration, duplex Western blotting was performed to detect simultaneously apo A-I and apo E in HDL on 2D-NDGGE. Our results clearly show that inhibition of CETP produces apo E-containing HDL particles almost completely devoid of apo A-I. The presence of these particles in human plasma was previously suggested (40, 43) but not confirmed due to the lack of simultaneous search for both apolipoproteins in previous studies. The mechanisms responsible for the appearance of these large apo E-rich HDL particles remain uncertain. HDL enrichment in cholesterol and phospholipids induced by CETP inhibition might favor the association of apo E to this lipoprotein, which eventually remodels into an apo A-I-free particle. Alternatively, apo E-rich HDL particles might have a cellular origin, since hepatocytes and monocyte-derived macrophages secrete a particle having the size of an HDL that contains mostly apo E (44).

In the absence of CETP-mediated cholesterol transfer from HDL to apo B-containing lipoproteins, apo E-rich HDL particles generated by CETPi could potentially play an important role in the direct pathway of RCT to the liver. Indeed, given the presence of apo E, a well-known ligand for members of the LDLr family of receptors (14, 15) and apo E-rich HDL particles could bind the LDLr or the LDLr-related protein in order to deliver cholesterol for clearance. To determine whether apo E-containing HDL particles have the capacity to associate with hepatic LDLr, we conducted a competition assay of fluorescent LDL association to HepG2 cells with apo B-depleted serum from treated rabbits. Our results showed that competition of LDL association is higher in the presence of serum from CETPi-treated rabbits than the corresponding baseline sera, while no significant difference was observed for control rabbits. Using HDL isolated from plasma by ultracentrifugation, we confirmed those results by a method separating lipoproteins according to their density rather than size as for PEG precipitation, in which large abnormal HDL could be precipitated. Moreover, we showed that incorporation of exogenous purified human apo E with human HDL or human apo-B-depleted plasma increases in a dose-dependent fashion the competition of DiI-LDL. Since the apo E concentration was chosen to correspond to the range of apo E increase measured in patients treated with dalcetrapib, these results strengthen the implication of the higher level of apo E in HDL in the increase of competition with LDL. These results suggest that apo E-rich HDL particles generated by CETPi have the capacity to associate with LDLr, in agreement with previous results showing that the latter receptor can mediate the binding of apo E-enriched HDL to human fibroblasts and rat liver (16, 17). This competition for the LDLr between apo E-containing HDL particles and LDL is also suggested by the demonstration that CETPi impeded the reduction of plasma LDL-C caused by atorvastatin. Indeed, enhanced competition of apo E-containing HDL for the LDLr may reduce LDL particle uptake in the liver and potentially increase LDL-C level. Accordingly, we observed an increase of cholesterol in isolated apo B-containing lipoproteins, which could be located in the more abundant VLDL particles of rabbits compared to LDL. Since no significant effect on TG level was observed, the apo E-enriched HDL may have a limited impact on TG-rich particle metabolism. Nevertheless, this increase of cholesterol in apo B-containing lipoproteins might counterbalance the potential benefits of the increase of HDL-C on cardiovascular diseases. In humans, dalcetrapib appears to have a similar effect, as suggested by the mean increase of large LDL particles at the expense of small LDL particle concentration. Given that small dense LDL was previously associated with cardiovascular disease, such a modulation of LDL subclass distribution may provide atheroprotection in patients (45). However, the increase of apo E caused by dalcetrapib may be partially responsible for the absence of significant LDL-C reduction (22). The evaluation of dalcetrapib’s effect on apo E modulation according to genotypes at the SNP rs1967309 in the *ADCY9* gene could contribute to the determination of the impact of apo E-containing HDL particles on cardiovascular endpoints. In that context, modulation of LDLr expression may greatly impact the cardiovascular effects of CETPi. Of interest, it was demonstrated that anacetrapib reduced the level of circulating PCSK9 in humans (46). This lower level of PCSK9 may increase LDLr expression and favor the clearance of apo E-containing HDL particles.

Apo E itself was linked to diverse atheroprotective mechanisms. Indeed, apo E was associated with the inhibition of inflammation in mouse macrophages RAW 264.7 stimulated with LPS (47) and inhibition of mouse aortic smooth muscle cell migration (48) and proliferation (49). Enrichment of plasma with apo E-containing HDL could mediate these effects in vivo as demonstrated by the enhancement of TNF-α mRNA reduction caused by apo B-depleted plasma from rabbits treated with atorvastatin in the presence of dalcetrapib or anacetrapib when compared to atorvastatin alone. Moreover, previous studies of apo E-containing HDL attributed various atheroprotective functions to these particles. Among others, it was shown that apo E-rich HDL reduced lipase-mediated retention of LDL by the subendothelial matrix (50). Results also demonstrated that apo E in HDL is required for binding to vascular proteoglycans, a mechanism potentially involved in the retention of HDL in the artery wall (51). Apo E-containing HDL particles have also been implicated in the inhibition of the expression of extracellular matrix genes by mouse smooth muscle cells (52). Moreover, it was demonstrated that plasma HDL containing only apo E was as effective as apo A-I in promoting cellular cholesterol efflux (44). Accordingly, we showed that cholesterol efflux capacity of apo B-depleted plasma from rabbits treated with atorvastatin in the presence of dalcetrapib or anacetrapib is increased compared to atorvastatin alone, suggesting that apo E-containing HDL produced by CETPi treatment have increased atheroprotective proprieties. Interestingly, it was shown that a higher ratio of apo E in HDL to total HDL-C is associated with a lower incidence of coronary heart disease in patients, suggesting that apo E-containing HDL exert cardioprotective effects (53).

Previous studies have demonstrated that statins reduce ABCA1 expression in human macrophages (54, 55). Given the crucial role of ABCA1 in cholesterol efflux and HDL biogenesis, we evaluated the impact of atorvastatin on the effects of dalcetrapib and anacetrapib. As demonstrated by the reduction of LDL-C, treatment with atorvastatin was effective in rabbits. HDL-C was also reduced by atorvastatin treatment, but this effect appears to be caused by the diet restriction more than a drug-dependent modulation as a similar reduction of HDL-C was noted in control animals. Moreover, as for the control group, this HDL-C reduction caused by atorvastatin had no significant impact on the increased HDL-C levels induced by CETPi. It was demonstrated in clinical studies that statins modestly increase the levels of HDL-C and apo A-I (56), suggesting that these agents have no major impact on efflux induced by HDL in vivo. Statins have also been shown to reduce the plasma level of apo E (18, 19, 20). Our results show that atorvastatin did not affect the increase of apo E concentration induced by CETPi in rabbits. Furthermore, the increase of apo E-containing HDL particles in the CETPi arm of the dal-PLAQUE-2 clinical trial suggests that statins do not prevent the effect of dalcetrapib on apo E in patients with CAD.

In summary, this study demonstrated that CETPi induce the formation of large apo E-containing HDL particles, devoid of apo A-I, that interact with the LDLr. The generation of these particles may be essential to RCT in the absence of CETP activity and of transfer of cholesterol from HDL to apo B-containing lipoproteins.

## Data availability

All data are contained within the article.

## Supplemental data

This article contains supplemental data.

## Conflict of interest

E. J. N. and C. M. were previously employees of F. Hoffmann-La Roche Ltd. J.-C. T. has received research grants from Amarin, AstraZeneca, Ceapro, DalCor Pharmaceuticals, Esperion, F. Hoffmann-La Roche Ltd., Ionis, Novartis, Pfizer, and RegenXBio; honoraria from AstraZeneca, DalCor Pharmaceuticals, HLS Pharmaceuticals, Pendopharm, and Pfizer; holds minor equity interest in DalCor Pharmaceuticals; and is mentioned as an author of a patent on pharmacogenomics-guided dalcetrapib treatment. The other authors declare that they have no conflicts of interest with the contents of this article.
