# Supplementary material for: Dalcetrapib and anacetrapib increase apolipoprotein E-containing HDL in rabbits and humans
Source: J Lipid Res. 2022 Nov 19;64(1):100316. doi: 10.1016/j.jlr.2022.100316 (PMC9793321; doi:10.1016/j.jlr.2022.100316)
Supplement: Supplemental Figures S1–S5 and Tables S1, S2 [file mmc1.pdf]

<u>**SUPPLEMENTAL INFORMATION**</u>

Dalcetrapib and anacetrapib increase apolipoprotein E-containing HDL in rabbits and humans.

Mathieu R. Brodeur[1,‡], David Rhainds[1,‡], Daniel Charpentier[1], Marie Boulé[1], Téodora Mihalache-Avram[1], Mélanie Mecteau[1], Geneviève Brand[1], Valérie Pedneault-Gagnon[1], Annik Fortier[3], Eric J. Niesor[4], Eric Rhéaume[1,2], Cyrille Maugeais[4] and Jean-Claude Tardif[1,2 *]

[1]Montreal Heart Institute, Montreal, Quebec, Canada; [2]Faculty of Medicine, Université de Montréal, Quebec, Canada; [3]Montreal Health Innovations Coordinating Center, Montreal, Quebec, Canada

[4]F. Hoffmann-La Roche Ltd, Basel, Switzerland

[‡] Contributed equally to the study

[*]To whom correspondence should be addressed: Jean-Claude Tardif, MD, Montreal Heart Institute Research Center, 5000 Belanger Street, Montreal, Canada, H1T 1C8.

Fax: 1-514-593-2500

Phone: 1-514-376-3330 ext.3604

e-mail: jean-claude.tardif@icm-mhi.org

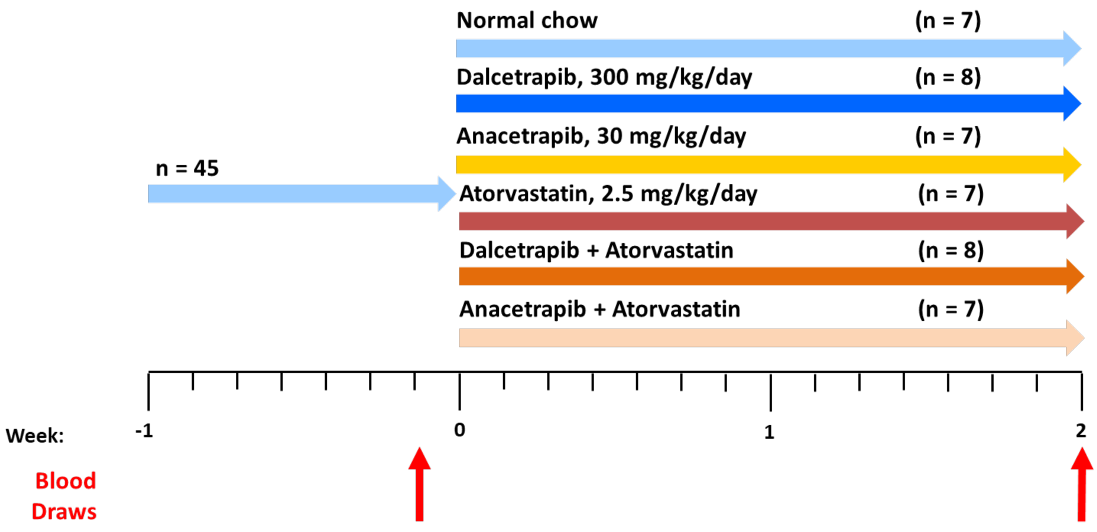

Supplemental Fig. S1: Schematic representation of experimental design of the rabbit study. Animals were acclimatized for 2 weeks under moderate caloric restriction. Then, rabbits were randomized according to their baseline HDL-C levels, to receive 300 mg/kg/day of dalcetrapib, 30 mg/kg/day of anacetrapib in presence or absence of 2.5 mg/kg of atorvastatin. Control group receiving chow diet or atorvastatin were also included in that study. Blood samples were obtained from the marginal ear vein 1 day before treatment start and on day 14 from animals fasted for ≥5 h.

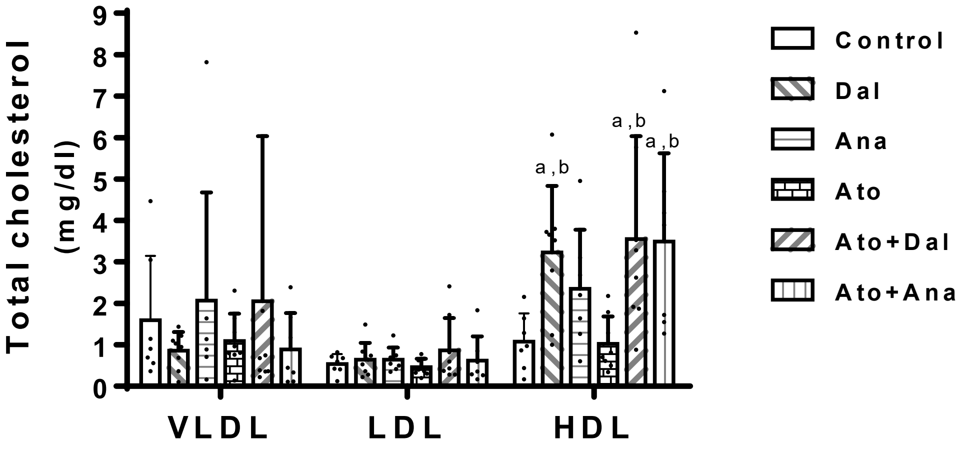

Supplemental Fig. S2. Impact of CETPi in absence or presence of atorvastatin on cholesterol content of lipoprotein classes separated by FPLC from rabbit plasma. Rabbits were treated with dalcetrapib (300 mg/kg) or anacetrapib (30 mg/kg) in presence or absence of atorvastatin (2.5 mg/kg). Plasma lipoprotein classes from day 14 samples were separated by FPLC and total cholesterol concentrations were obtained in each fraction. Cholesterol associated with VLDL, LDL and HDL was evaluated by the sum of cholesterol detected in fraction 8-12, 13-18 and 19-28, respectively. Results are presented as mean ± SD of n=7-8 rabbits. One-way ANOVA was used for statistical analysis. [a] different from control group, [b] different from atorvastatin group.

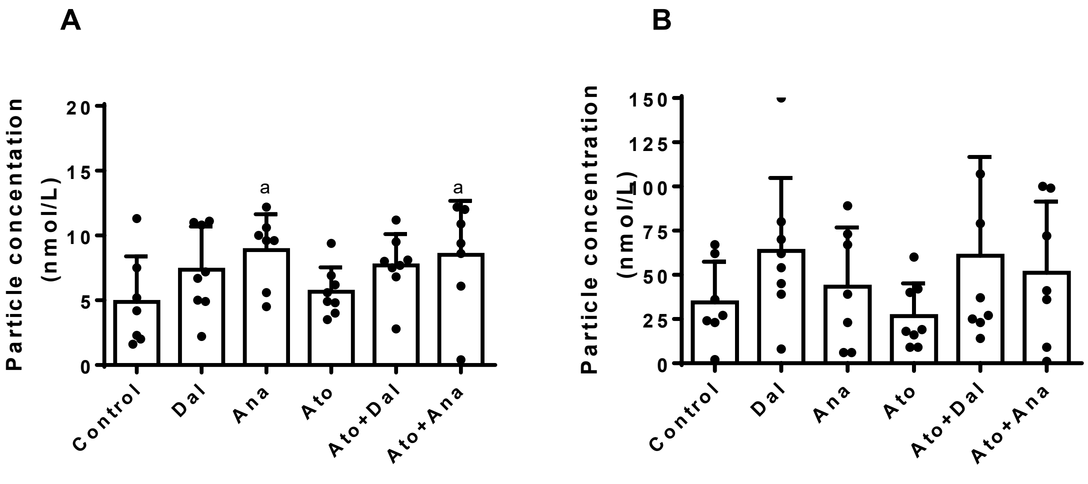

Supplemental Fig. S3. Impact of CETPi in absence or presence of atorvastatin on the lipoprotein particle concentrations measured by nuclear magnetic resonance (NMR). Rabbits were treated with dalcetrapib or anacetrapib in presence or absence of atorvastatin. At day 14, large HDL (A) and LDL (B) concentrations were measured by NMR. Results are presented as mean ± SD of 7 to 8 animals. One-way ANOVA was used for statistical analysis. [a] different from control group.

A

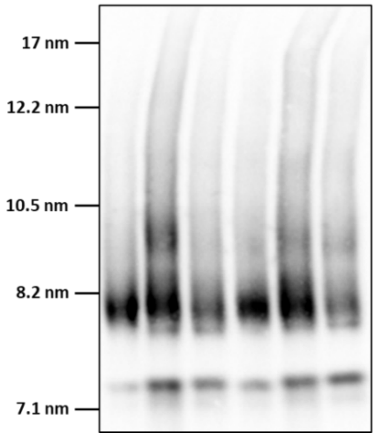

B

	Small HDL % (7.1 nm – 8.8 nm)	Large HDL % (8.8 nm – 12.2 nm)
Control	62.3	37.7
Dal	49.7	50.3
Ana	58.5	41.5
Ato	60.0	40.0
Ato+Dal	52.0	48.0
Ato+Ana	53.8	46.2

Supplemental Fig. S4. Impact of CETPi in absence or presence of atorvastatin on HDL subclasses percentages. Rabbits were treated with dalcetrapib or anacetrapib in absence or presence of atorvastatin and HDL were isolated by ultracentrifugation from a pool of plasma and migrated on 4-30% one-dimensional non-denaturating gradient gel electrophoresis (1D-NDGGE). (A) Representative western blot of apo A-I on isolated rabbits HDL and (B) percentage of small and large HDL calculated by densitometric analysis of western blot.

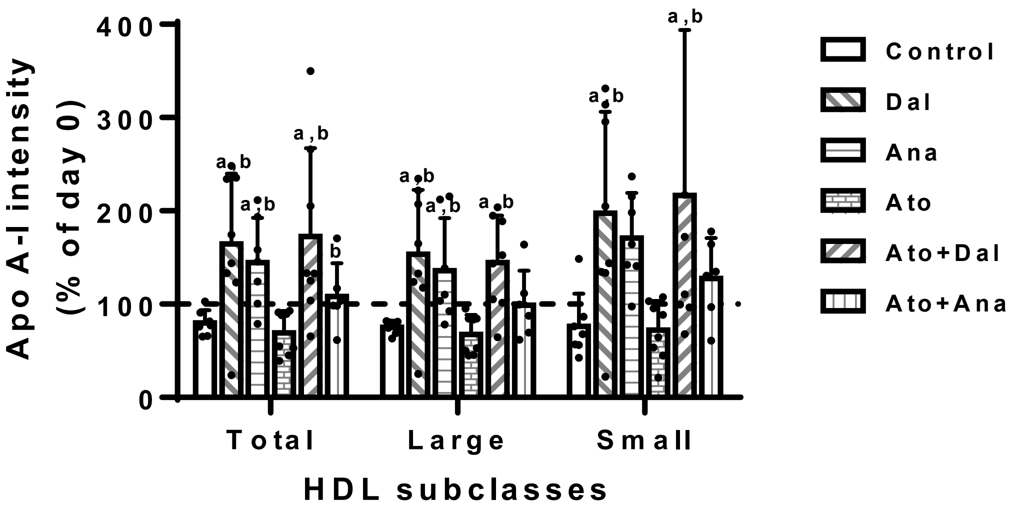

Supplemental Fig. S5. Impact of CETPi in absence or presence of atorvastatin on rabbit apo A-I distribution in HDL subclasses. Rabbits were treated with dalcetrapib or anacetrapib in presence or absence of atorvastatin. At day 0 and 14, plasma was harvested and used for 4–30% 1D-NDGGE before densitometric analysis of apo A-I Western blots. Percentage was calculated from band intensity of large (8.8-12.2 nm) and small (7.1-8.8 nm) HDL at day 14 and day 0 for each rabbit. Results are presented as mean ± SD of 7 to 8 animals. One-way ANOVA was used for statistical analysis. [a] $P<0.05$ different from control group and [b] $P<0.05$ different from atorvastatin group.

Supplemental Table S1. Sequences of primers used for quantification of TNF-α mRNA expression by reverse transcription-quantitative PCR.

	**Forward (5'-3')**	**Reverse (5'-3')**
SDHA	GAGAACAAGAAGGCATCA	CTACAACCACAGCATCAA
B2M	GCCTGTATGCTATCCAGAA	TCAGTATGTTCGGCTTCC
TNF-α	GGTTCTGTCCCTTTCACTCAC	CTCTTCTGCCAGTTCC

Supplemental Table S2. Impact of CETPi in absence or presence of atorvastatin on the level of apolipoprotein E, C-I and C-III peptides associated with isolated HDL measured by liquid-chromatography-mass spectrometry (LC-MS/MS).

	**Apolipoprotein E**	**Apolipoprotein C-I**	**Apolipoprotein C-III**
**Control**	6357	4662	12 502
**Dal**	10 640	10 640	18 620
**Ana**	7506	5004	13 761
**Ato**	4851	3234	6738
**Ato+Dal**	9435	8301	15 847
**Ato+Ana**	10 244	8478	16 603

Rabbits were treated with dalcetrapib or anacetrapib in presence or absence of atorvastatin. HDL from pooled plasmas (day 14) of each group was isolated by ultracentrifugation and the number of apo E peptides was measured by LC-MS/MS. Results are presented as the number of apolipoprotein peptides found in the total amount of isolated HDL.
